# Noninvasive Brain Stimulation for Nicotine Dependence in Schizophrenia: A Mini Review

**DOI:** 10.3389/fpsyt.2022.824878

**Published:** 2022-02-09

**Authors:** Heather Burrell Ward, Roscoe O. Brady, Mark A. Halko, Paulo Lizano

**Affiliations:** ^1^Department of Neurology, Berenson-Allen Center for Noninvasive Brain Stimulation, Beth Israel Deaconess Medical Center, Boston, MA, United States; ^2^Department of Psychiatry, Beth Israel Deaconess Medical Center and Harvard Medical School, Boston, MA, United States; ^3^McLean Hospital, Belmont, MA, United States; ^4^Harvard Medical School, Boston, MA, United States

**Keywords:** schizophrenia, substance use disorder (SUD), nicotine dependence, smoking, noninvasive brain stimulation (NIBS), repetitive transcranial magnetic stimulation (rTMS), transcranial direct current simulation, tobacco

## Abstract

Individuals with schizophrenia are 10 times more likely to have a tobacco use disorder than the general population. Up to 80% of those with schizophrenia smoke tobacco regularly, a prevalence three-times that of the general population. Despite the striking prevalence of tobacco use in schizophrenia, current treatments are not tailored to the pathophysiology of this population. There is growing support for use of noninvasive brain stimulation (NIBS) to treat substance use disorders (SUDs), particularly for tobacco use in neurotypical smokers. NIBS interventions targeting the dorsolateral prefrontal cortex have been effective for nicotine dependence in control populations—so much so that transcranial magnetic stimulation is now FDA-approved for smoking cessation. However, this has not borne out in the studies using this approach in schizophrenia. We performed a literature search to identify articles using NIBS for the treatment of nicotine dependence in people with schizophrenia, which identified six studies. These studies yielded mixed results. Is it possible that nicotine has a unique effect in schizophrenia that is different than its effect in neurotypical smokers? Individuals with schizophrenia may receive additional benefit from nicotine's pro-cognitive effects than control populations and may use nicotine to improve brain network abnormalities from their illness. Therefore, clinical trials of NIBS interventions should test a schizophrenia-specific target for smoking cessation. We propose a generalized approach whereby schizophrenia-specific brain circuitry related to SUDs is be identified and then targeted with NIBS interventions.

## Introduction

Worldwide, 1.3 billion people use tobacco (1). Individuals with schizophrenia are 10 times more likely to have a tobacco use disorder than the general population (2–4). It is estimated that 64–79% of those with schizophrenia smoke tobacco regularly (5, 6), a prevalence three-times that of the general population. As a result, people with schizophrenia die nearly 30 years earlier from illnesses attributable to tobacco smoking (7).

Noninvasive brain stimulation (NIBS) has been investigated for the treatment of substance use disorders (SUDs), including nicotine dependence. Repetitive transcranial magnetic stimulation (rTMS) and transcranial electrical stimulation (tES) are the two most common forms of NIBS being investigated for SUDs. In the application of rTMS, an electromagnetic coil is placed on the scalp. An electrical current is pulsed through this wire coil, which generates a magnetic field that can either increase or decrease neuronal firing beneath the coil. Multiple pulses are delivered at a given frequency, intensity, and duration. These parameters change the neuronal effects of rTMS. High frequency rTMS (e.g., 10-20 Hz) tends to increase neuronal firing, while low frequency rTMS (e.g., 1 Hz) tends to decrease neuronal firing (8).

tES influences brain circuits by producing a weak direct or alternating current through the use of electrodes placed over the scalp. The electrical currents from tES facilitate action potentials, where anodal stimulation enhances cortical excitability and cathodal stimulation diminishes cortical excitability (9–12). Although there are different types of tES, we focus on transcranial direction current stimulation (tDCS), which involves a continuous source of electrical stimulation and is non-frequency dependent (13). In tDCS, a low intensity direct current is applied to the scalp using two or more electrodes. Under the anodal electrode, resting membrane potential decreases, which increases cortical excitability, while under the cathodal electrode, the membrane is hyperpolarized, which decreases excitability (9–11).

**Figure 1:**
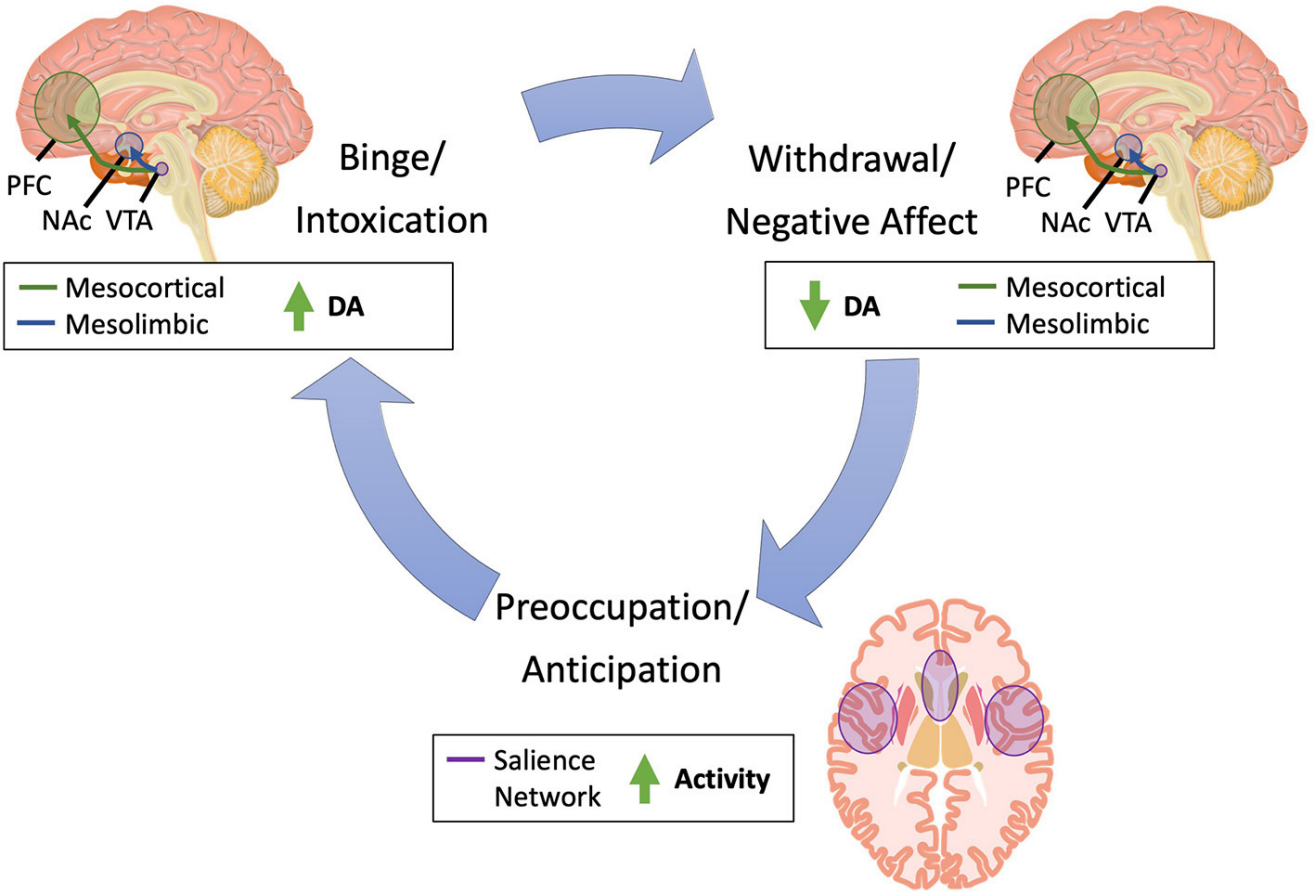
Brain pathways implicated in substance use. Substance use is characterized by three primary phases: (1) binge/intoxication, (2) withdrawal/negative affect, and (3) preoccupation/anticipation (14). In the binge/intoxication phase, substance use leads to acute increases in dopamine in the mesolimbic and mesocortical circuitry, which drives incentive salience and drug-seeking. In the withdrawal/negative affect, dopamine acutely decreases in these same circuits, leading to negative emotional states and dysphoric and stress responses. Finally, in the preoccupation/anticipation phase, increased activity in the salience network, which includes the insula and dorsal anterior cingulate cortex, leads to craving and deficits in executive function. DA, dopamine; NAc, nucleus accumbens; PFC, prefrontal cortex; SN, substantia nigra; VTA, ventral tegmental area.

NIBS techniques enable targeted intervention on specific brain circuits, including those involved in the development and persistence of SUDs (Figure 1). The largest body of evidence supports the use of NIBS for tobacco use, as evidenced by the August 2020 Food and Drug Administration approval of rTMS for smoking cessation (15).

Despite the problem of tobacco use in schizophrenia, there have been very few studies of NIBS in this population. In this Mini-Review, we briefly review the neurobiological evidence supporting NIBS for nicotine dependence in a non-schizophrenia population followed by the existing literature using NIBS for nicotine dependence in schizophrenia. We consider the mixed results of the trials of NIBS for nicotine dependence in schizophrenia and conclude by offering a novel path forward whereby schizophrenia-specific brain circuitry related to SUDs is be identified and then targeted with NIBS interventions.

### NIBS for Nicotine Dependence Targets the Dorsolateral Prefrontal Cortex

Multiple studies support the use of rTMS for nicotine dependence in healthy smokers (15–21). Most of these studies have used high frequency 10 Hz rTMS delivered to the left dorsolateral prefrontal cortex (DLPFC) ranging from a 15-min single-session (17) to multi-session experiments (15, 16, 19, 21). High frequency stimulation of the DLPFC has been proposed to activate this region and thereby improve “top-down” regulation of brain regions involved in craving and drug-seeking behavior (22). Moreover, administration of rTMS to the left DLPFC stimulates dopamine release in the striatum (23, 24), anterior cingulate cortex, and medial prefrontal cortex (24).

However, in the Zangen et al. study, which earned rTMS FDA-approval for smoking cessation, high frequency bilateral rTMS was targeted to the lateral prefrontal and insular cortices (15). This study used an H4-coil (Brainsway, Israel), which has been shown in electric field models to bilaterally stimulate neuronal pathways in the lateral prefrontal cortex and insula at an intensity above the neuronal threshold for activation. Targeting of these regions has been proposed to reduce craving in response to smoking cues.

Active rTMS treatment has been associated with decreased cigarette consumption and craving compared to sham (15–17, 19, 21). Moreover, a systematic review and meta-analysis observed that 10 Hz rTMS to the left DLPFC was associated with the greatest reductions in smoking frequency (25).

Despite these promising clinical data, the mechanism of the effects of rTMS on circuits related to nicotine dependence remains largely unknown. Li et al. observed that after 10 sessions of 10 Hz rTMS to the left DLPFC, active TMS inhibited brain activity in the right insula and thalamus and decreased connectivity from the DLPFC to the left medial orbitofrontal cortex, suggesting TMS may reduce reactivity to smoking cues (18).

In addition to rTMS, tDCS has also been shown to impact nicotine dependence. Anodal DLPFC (left and right) tDCS has also demonstrated effectiveness in reducing cue-induced craving and cigarette consumption (26–28). A systematic review and meta-analysis observed that right-anodal, left-cathodal tDCS to the DLPFC significantly reduced cue-induced craving (29). Another study observed that 20 sessions of tDCS over 12 weeks for smoking cessation achieved comparable abstinence rates as 8 weeks of treatment with 300 mg bupropion (30). Despite multiple studies supporting the effects of tDCS on tobacco use, another group observed the effects of tDCS on cigarette craving, cigarette consumption, and executive function were no different from placebo (31).

The mechanism by which tDCS affects craving and cigarette use also remains unknown. Anodal right DLPFC/cathodal left occipital tDCS reduced smoking craving and increased brain reactivity to smoking cues in the right posterior cingulate (32). There is evidence linking the effects of tDCS to the DMN in smokers. Left-anodal DLPFC/right-cathodal vmPFC tDCS increased deactivation of DMN nodes during a working memory task and increased anterior cingulate activity during an error-monitoring task. The effect of tDCS on the DMN was more pronounced when smokers were in a sated (rather than withdrawn) state (33).

### NIBS for Nicotine Dependence in Schizophrenia Has Mixed Results

We performed a literature search to identify articles using any form of NIBS for the treatment of nicotine dependence in people with schizophrenia. We searched PubMed using search terms for NIBS (i.e., transcranial electrical stimulation, transcranial magnetic stimulation, transcranial direct current stimulation, transcranial alternating current stimulation), schizophrenia, and nicotine dependence. We excluded any articles that were not primary research studies, including literature reviews, case reports, and meta-analyses.

Studies not meeting inclusion criteria were excluded based on title and abstract. The remaining studies were evaluated based on full-text articles and selected if they met inclusion and exclusion criteria. Following this initial search and screen, we manually searched any relevant systematic reviews and performed a citation analysis to identify any additional articles that met inclusion criteria.

Our initial search identified 21 results. After screening titles and abstracts, 13 full-text manuscripts were evaluated. Seven of these articles were excluded for not studying schizophrenia (*n* = 1), not investigating NIBS as a treatment for nicotine dependence (*n* = 3), and not primary research articles (*n* = 3). We identified six studies of NIBS interventions for nicotine dependence in schizophrenia that met our inclusion and exclusion criteria (Table 1 and Supplementary Figure 1).

**Table 1:** Comparison of studies using NIBS for nicotine dependence in schizophrenia.

**References**	**TMS/tDCS**	***N***	**Smoking cessation treatment**	**Stimulation site**	**Parameters**	**Sessions**	**Sham-controlled?**	**Craving**	**Cigarette use**
Wing et al. (34)	TMS	15	Nicotine patch (21 mg)	Bilateral DLPFC	20 Hz, 90% RMT, 750 pulses	20	Yes	**↓**	No change
Prikryl et al. (35)	TMS	35	-	L DLPFC	10 Hz	21	Yes	-	**↓**
Huang et al. (36)	TMS	37	-	L DLPFC	10 Hz	21	Yes	-	**↓**
Kamp et al. (37)	TMS	67	-	L DLPFC	10 Hz	15	Yes	-	No change
Kozak et al. (38)	TMS	27	-	Bilateral DLPFC	20 Hz	6	No	No change	-
Smith et al. (39)	tDCS	37	-	Anode L DLPFC; Cathode contralateral supraorbital ridge	2 mA	5	Yes	No change	No change

*DLPFC, dorsolateral prefrontal cortex; -, not assessed*.

The studies were from multiple countries, including Canada (*n* = 2) (34, 38), China (*n* = 1) (36), the Czech Republic (*n* = 1) (35), Germany (*n* = 1) (37), and the United States (*n* = 1) (39).

Of the six studies we identified, there were five studies of rTMS and one study of tDCS. Five studies were randomized, sham-controlled trials. One study involved only open-label treatment. Only two studies observed a decrease in cigarette use while only one study observed decreased cigarette craving. We describe these studies in detail in order to understand the reason for these mixed results.

We identified 5 studies using rTMS for nicotine dependence in schizophrenia (34–38). Wing et al. conducted a 10-week randomized, double-blind, sham-controlled trial of rTMS for smokers with schizophrenia to reduce craving during a smoking cessation attempt (34). Individuals received 4 weeks of rTMS (Weeks 1-4) in addition to weekly group therapy and transdermal nicotine patch (21 mg) (Weeks 3-9). High frequency rTMS (20 Hz) was delivered bilaterally to the DLPFC five times per week for a total of 20 sessions. Fifteen participants were randomized to active stimulation (*n* = 6) or sham stimulation in the single-wing tilt position (*n* = 9). Active treatment with rTMS significantly reduced craving in week 1 but not in weeks 2-4. Notably, rTMS did not increase abstinence rates. Authors suggested that future studies should evaluate rTMS for smoking cessation in the absence of therapy and nicotine patch in a larger study population and after a longer period of abstinence.

In a secondary analysis of the rTMS for the Treatment of Negative Symptoms in Schizophrenia (RESIS) trial, Kamp et al. analyzed the effect of high frequency rTMS on daily cigarette consumption in a sample of individuals with schizophrenia with predominantly negative symptoms (37). Participants (*n* = 67) were randomized to active or sham rTMS. Active rTMS (10 Hz) was administered to the left DLPFC five times per week for 3 weeks for a total of 15 sessions. Investigators did not observe a significant effect of time, group, or group × time on daily cigarette consumption.

Kozak et al. also delivered 20 Hz rTMS to the left DLPFC but instead used a crossover study design (38). Participants received twice daily rTMS for 3 days. Individuals were assessed under conditions of nicotine satiety (day 2), following 16 h of acute abstinence (day 3 morning), and upon smoking reinstatement (day 3 afternoon). A total of 27 participants (13 schizophrenia, 14 controls) completed the study. Investigators observed that overnight abstinence produced the expected effects of increasing tobacco craving and withdrawal and impairing cognitive performance. However, active rTMS did not affect this pattern, suggesting that 3 days of rTMS was insufficient to reduce the acute effects of nicotine withdrawal.

Prikryl et al. delivered 10 Hz rTMS or sham to the left DLPFC in 35 male schizophrenia participants for 21 days (35). They observed that cigarette consumption was significantly reduced for the active treatment group after only 1 week of stimulation. This reduction remained statistically significant through the follow-up assessment.

Huang et al. applied 10 Hz rTMS or sham to the left DLPFC in 37 non-treatment-seeking male smokers with schizophrenia for 21 days. Individuals who received active rTMS showed a significant reduction in number of daily cigarettes smoked beginning after the first week of treatment (36). This significant reduction in cigarette use was sustained in the rTMS group compared to the control group through the follow-up assessment 21 days after treatment ended. There were no correlations between reduction in cigarette use and schizophrenia symptoms, depressive symptoms, or performance on the Wisconsin Card Sorting Test.

We identified only one study of tDCS for nicotine use in schizophrenia (39). Smith et al. applied 2 mA tDCS for five sessions to 37 individuals with schizophrenia. They observed the active treatment group had significant improvements in the MATRICS Consensus Cognitive Battery composite score and subscores for working memory and attention-vigilance. However, they did not observe any significant changes in psychiatric symptoms, cigarette consumption, or craving.

In summary, only half of these studies reported a significant effect on cigarette use or craving in schizophrenia. What explains the mixed results when NIBS is applied to the DLPFC in schizophrenia?

### Is There an Alternative Network and Cognition-Centric Explanation for the Smoking Prevalence in Schizophrenia?

NIBS targeting the DLPFC has been effective for nicotine dependence in neurotypical smokers—so much so that it is now an FDA-approved treatment for smoking cessation. However, this has not borne out in the studies using this approach in schizophrenia. Is it possible that nicotine has a unique effect in schizophrenia? If that is the case, then perhaps NIBS interventions should instead be tested on a schizophrenia-specific target.

Individuals with schizophrenia may receive additional benefit from nicotine's pro-cognitive effects than control populations. Nicotine improves cognition both in controls and in individuals with schizophrenia (40). Nicotine's pro-cognitive effects are largely due to binding to the alpha7 subunit of the nicotinic acetylcholine receptor (nAChR) in the hippocampus and anterior cingulate (41, 42). In schizophrenia, this leads to improved sensory gating, improved attention (43) and working memory (44), and increased thalamocortical functional connectivity (45). Individuals with schizophrenia have decreased nAChR expression in brain regions that are central to higher cognitive functioning (46). Moreover, following nicotine withdrawal, schizophrenia individuals show greater impairments in attention and executive function than healthy controls (47).

Nicotine's effects on cognition have been linked to reducing the activity and connectivity of the default mode network (DMN). The DMN is active during self-referential thinking (48, 49). Importantly, DMN activity is suppressed when one is engaging in a task, and task performance is dependent upon successfully suppressing DMN activity. Impaired attention has been linked to DMN hyperconnectivity in healthy controls (50) and schizophrenia (51, 52). Acute nicotine administration in healthy controls decreases DMN activity during an attention task (53) and in the resting state (54). Nicotine suppresses activity in the DMN while withdrawal activates it (55, 56). Moreover, during nicotine withdrawal, the DMN has been observed to be hyperconnected (57). The DMN is activated during exposure to smoking-related cues (58–60).

People with schizophrenia may use nicotine in order to improve brain network abnormalities from their illness. Nicotine's cognitive-enhancing effects have been linked to reduction of DMN activity and hyperconnectivity. Notably, DMN hyperconnectivity is a hallmark of the neurobiology of schizophrenia (61). Therefore, individuals with schizophrenia may be using nicotine as a form of “self-medication” in order to reduce their default mode network hyperconnectivity and thereby improve their cognitive performance. This would imply a schizophrenia-specific brain basis for the pathophysiology of nicotine dependence in this population and would therefore suggest NIBS interventions should use an alternative target in schizophrenia.

## Discussion

The prevalence of nicotine dependence in schizophrenia is staggering compared to the general population. Despite the significant decreased life expectancy caused by tobacco use in this population, there are no schizophrenia-specific smoking cessation treatments.

NIBS is being investigated for multiple SUDs, including nicotine dependence. rTMS recently received FDA-approval as a treatment for smoking cessation in neurotypical smokers. We identified 6 studies of NIBS for nicotine dependence in schizophrenia. These studies all stimulated the DLPFC, with the goal of improving “top-down” regulation of brain circuitry involved in reward and response to smoking cues (i.e., salience). However, their results were heterogeneous, suggesting the same target used to treat nicotine dependence in controls may not be effective in schizophrenia.

This suggests there is perhaps an alternative explanation for the etiology of nicotine dependence in schizophrenia. Accordingly, this would also suggest a schizophrenia-specific target should be identified for NIBS interventions.

We would propose that the DMN may be a schizophrenia-specific target for nicotine dependence in schizophrenia. Nicotine has been linked to improved attentional performance, and impaired attentional performance is associated with DMN hyperconnectivity, a finding commonly observed in schizophrenia. Acute nicotine administration reduces this DMN hyperconnectivity. This suggests individuals with schizophrenia may be using nicotine to reduce the hyperconnectivity of their DMN in order to improve cognitive deficits. Therefore, TMS could be used to restore normal connectivity patterns in the DMN, potentially improving cognitive performance and reducing the drive to use nicotine in schizophrenia. In this way, the DMN could offer a schizophrenia-specific target for NIBS smoking cessation treatments. Clinical trials targeting the DMN for smoking cessation in schizophrenia are readily accessible with existing technology and should be conducted. Indeed, previous studies have modulated the DMN by stimulating network nodes in the cerebellum in healthy controls (62) and in schizophrenia (63).

NIBS interventions offer great potential to develop treatments for other co-occurring substance use disorders in schizophrenia. In order to develop such treatments, we must first identify the brain circuit abnormalities unique to co-occurring substance use and schizophrenia, similarly to what we have proposed with nicotine dependence. NIBS interventions can be used to perturb the identified neurocircuitry and measure changes in substance use outcomes (e.g., subjective substance use, biochemical measures of substance use, craving). This process thereby allows for identification of causal relationships between brain circuitry and substance use patterns (64). Then, forms of NIBS can be developed to target these abnormal brain circuits as substance use interventions.

## Author Contributions

All authors contributed to the conception and preparation of the manuscript and approved of the final version.

## Funding

This work was supported by the Sidney R. Baer, Jr. Foundation (HW), the Harvard Medical School Norman E. Zinberg Fellowship in Addiction Psychiatry Research (HW), NIMH R01MH116170 (RB), NIMH R01MH111868 (MH), NIMH R56MH125995 (MH), and NIH UL1 TR002541 (PL). Funding agencies had no input into manuscript conception or preparation.

## Conflict of Interest

The authors declare that the research was conducted in the absence of any commercial or financial relationships that could be construed as a potential conflict of interest.

## Publisher's Note

All claims expressed in this article are solely those of the authors and do not necessarily represent those of their affiliated organizations, or those of the publisher, the editors and the reviewers. Any product that may be evaluated in this article, or claim that may be made by its manufacturer, is not guaranteed or endorsed by the publisher.
