# Supplementary material for: Noninvasive Brain Stimulation for Nicotine Dependence in Schizophrenia: A Mini Review
Source: Front Psychiatry. 2022 Feb 9;13:824878. doi: 10.3389/fpsyt.2022.824878 (PMC8863675; doi:10.3389/fpsyt.2022.824878)
Supplement: Supplementary file 1 [file Data_Sheet_1.docx]

**Supplement**

**Supplemental Figure 1. Study Evaluation Diagram**


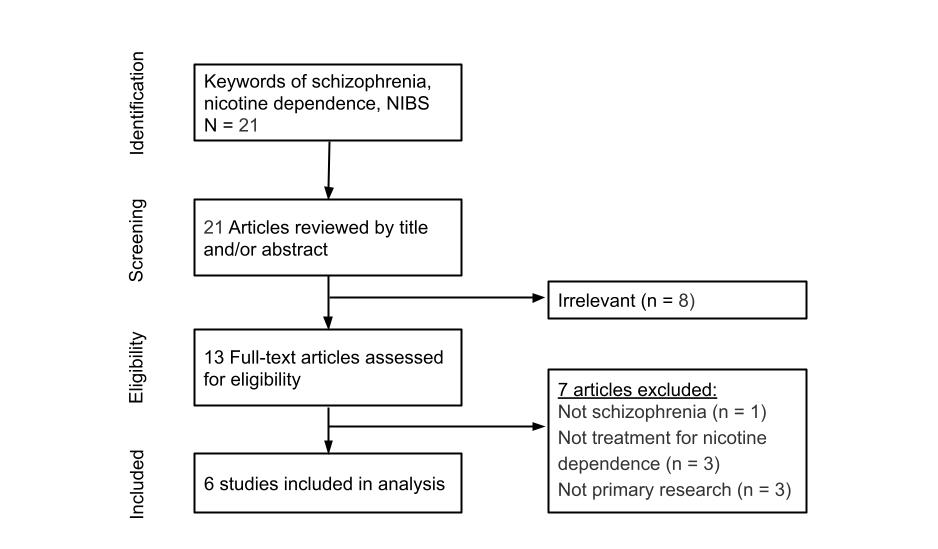


We performed a literature search to identify articles using any form of NIBS for the treatment of nicotine dependence in people with schizophrenia. We searched PubMed using search terms for NIBS (i.e. transcranial electrical stimulation, transcranial magnetic stimulation, transcranial direct current stimulation, transcranial alternating current stimulation), schizophrenia, and nicotine dependence. We excluded any articles that were not primary research studies, including literature reviews, case reports, and meta-analyses. Our initial search identified 21 results. After screening titles and abstracts, 13 full-text manuscripts were evaluated. Seven of these articles were excluded for not studying schizophrenia (n = 1), not investigating NIBS as a treatment for nicotine dependence (n = 3), and not primary research articles (n = 3). We identified 6 studies of NIBS interventions for nicotine dependence in schizophrenia that met our inclusion and exclusion criteria.
